# Dental Assertion and Discussion

**Published:** 1889-09

**Authors:** Joseph Head

**Affiliations:** Philadelphia


### DENTAL ASSERTION AND DISCUSSION.[1]

BY JOSEPH HEAD, D.D.S., PHILADELPHIA.

Many eminent members of the profession have remarked and deplored the inaccuracies that so frequently characterize the general atmosphere of dental conventions. And although I wish that some one with more power than I possess had undertaken to discuss this subject, yet its importance demands consideration, as it has to do with the advancement or deterioration of dentistry. I stand before you to-day, not with the idea of stating anything new or startling, but solely for the purpose of showing the superiority of demonstrated scientific fact over mere verbal assertion.

If assertions are properly supported by conclusive proof, then discussion becomes in the highest degree instructive; but statements that may or may not arise from personal prejudice can at best only point in the direction where truth may lie.

In the scientific world, no single man's opinion can be taken as conclusive. The accuracy of all experiments is doubted until the work has been confirmed again and again. For instance, if an investigator declares that he has discovered a new law, he is expected to publish a minute description of all the experiments pertaining to the establishment of that law. These experiments are thoroughly tested by his fellow-scientists, and if the deductions published are found to be correct, he is credited with a discovery. But if the experiments are found to have been erroneous or the deductions incorrect, the would-be discoverer gains a reputation for carelessness only.

Were not deductions compelled to undergo this severe scrutiny, it is quite evident that scientific literature would soon be thoroughly contaminated.

Such caution not only protects against wilful misrepresentation, but it also excludes all mistakes that may arise from inefficiency or heedlessness.

The need for these precautions becomes painfully evident, when we remember a certain animated discussion that has taken place within the last nine months on the subject of root-canal fillings. Gold, gutta-percha, oxychloride of zinc, cotton, and air were re-

---

[1] Read at the twenty-first annual meeting of the Pennsylvania State Society, held at Cresson, July 30, 1889.

spectively advocated. Each speaker in the debate decried all who differed in opinion from himself, and yet each failed to substantiate his assertions either by experimental or statistical proof.

Discussion carried on in such a loose manner must necessarily prove very confusing to young and inexperienced dentists, who of course attend these meetings for the purpose of receiving enlightenment from their fellow-practitioners; but flooded by a stream of unsupported statement, these bewildered young men find no solid foundation on which to base an opinion. So it happens that instead of being stimulated to careful investigation, they are for the most part terrified by numbers, overawed by superior age and reputation, and expend their valuable energies in shouting with the majority.

How different the result might be, we can readily imagine, if the disputants had been compelled to demonstrate the truth of their assertions by experiments on the extracted tooth, since a clear demonstration would have proved a basis for logical deduction.

If proofs are not required they will probably not be given, as most men, even unconsciously, are prone to describe their own discoveries as just a little better than they are.

Hearsay assertions and statements founded on practice work quite as much for harm as for good, since they merely give additional authority to suppositions that may be intrinsically false.

In order that we may guard ourselves against error, let us insist that each person taking part in a discussion be able to prove his assertions either by experiments, the accuracy of which can be tested, or by statistics which contain the dates and clear descriptions of all the operations mentioned. Had this rule been in operation at the last Dental Convention held by the Odontological Society of Philadelphia, the remarks of certain gentlemen would have been much more instructive. The subject of root-canal fillings was under discussion. One member said that he always measured the size of the apical foramen. Then he ascertained the exact length of the canal. This being accomplished, he placed a piece of gold on the end of his root-plugger, and, carrying it along the canal, filled the foramen, so that no moisture could possibly enter.

This, gentlemen, was an easy assertion to make, but I regret to say that some dentists present doubted his power to fill the roots of molars and bicuspids with the degree of exactness which he claimed. But consider what a death-blow he would have given to all scepticism had he supplemented his statement by continuing as follows: "In proof of my assertion, gentlemen, I bring this tooth, which has been filled in the following manner. But first, let me say that I have chosen a molar, because, as you know, molars are by far the most difficult of all the teeth to treat, and also the most important, as they constitute by far the largest class. With this slight break, permit me to explain how I filled the roots of this twelfth-year molar. I first placed the tooth in plaster in order that I might not be guided by the length and shape of its roots. Then, by means of the delicacy of my touch, I enlarged the tortuous canals, being especially careful to measure the apical foramen of the anterior buccal root. Lastly, as I have before stated, I packed in my gold so as absolutely to seal the tips of the roots. In proof of this assertion, examine the exposed canals and you will find hard, dense gold completely closing the foramen of all the roots."

Fellow-practitioners, when the speaker referred to neglected to bring forward such simple proof, he lost an opportunity for gaining immortal fame.

But to continue. Another speaker arose, who began by saying that all the dentists in the Eastern States were in the dark ages; that they were at least twenty-five years behind the times; that he agreed with the gentleman who filled roots with gold, but thought it better first to pump the canals full of liquid gutta-percha, afterwards packing in the gold as accurately as possible. In this way he was doubly sure of a tight joint.

Now, some of his colleagues might have replied that in his method the gold occupied the position of a useless mass only, while gutta-percha, weakened by chloroform, was the sole bar to the entrance of septic matter. And, moreover, they might have added that gutta-percha is a leaky canal-filling at best.

But what could the doubters have answered had he risen and spoken as follows: "Gentlemen, here is a molar tooth, the roots of which have first been imbedded in plaster, and then filled according to my method. I have soaked the tooth in red aniline ink for two months. Examine the canals and you will find the foramina free from stain." His proof would have been unanswerable, that is to say, if his experiments should have withstood investigation.

This same gentleman claimed that he saved seventy-five per cent. of all the pulps he capped. Here statistics would have been valuable. Had he shown a record of but two hundred cases where three-fourths of the pulps capped had lived three years, his remarks would have been much more impressive and less open to silent criticism.

Gentlemen, I might go on citing case after case, but it would be useless. We all appreciate the need for reform. If dentistry is to take her rightful place in the world of science, her sons must make their experiments more carefully and report their deductions with a greater degree of precision. Dentistry should be the most accurate of all the healing sciences, as it is most capable of practical demonstration; because out of the body the teeth, in many instances, can be used for experiment even more efficiently than when they are *in situ*, since, when the experiment is completed on the extracted tooth, the tooth can be divided into sections, thereby demonstrating the success or failure of the investigator's efforts.

These facts being accepted, it would seem that all dentists claiming to have a remarkable degree of success or skill in performing purely mechanical operations on the teeth should be required to prove their assertions on teeth whose roots have been buried in plaster. When they treat of pain or inflammation, carefully-prepared statistics should be required. These statistics would be extremely valuable, for when the records of three or four dentists pointed to the same conclusion, the proof would be good that the conclusion was correct.

Gentlemen, I do not mean to say that single incidents of practice should not be reported, nor would it be advisable that a dentist should refrain from giving his personal opinion. But it must be clear to all that no great law for the guidance of the profession should be deduced from one or two cases. And when an opinion is offered, let it be as an opinion, and not as an absolute assertion. Office experience, unsustained by statistics or experiments, reminds one of a certain little boy who rushed into his father's study, shouting, "Father, there are three thousand cats in the back yard." "Come," replied his father, "how many cats are there?" "Well, there's a hundred, or, at any rate, our old Tom and another."

To conclude: Brother practitioners, in our dental conventions, let us always remember the celebrated maxim of George Washington,—"In company never tell things which are hard to believe" (especially without substantial proofs).

